# Call for Adoption of Synchronized Biweekly Dosing of Anti-EGFR Agent Cetuximab: Implications for Patients with Metastatic Colorectal Cancer, and Squamous Cell Carcinoma of the Head and Neck

**DOI:** 10.1093/oncolo/oyac070

**Published:** 2022-04-11

**Authors:** Pashtoon Murtaza Kasi

**Affiliations:** Weill Cornell Medicine, Meyer Cancer Center, Englander Institute of Precision Medicine, 1305 York Ave, New York, NY, USA

## Abstract

This commentary highlights the article by Parikh and colleagues, regarding biweekly therapy with the anti-EGFR monoclonal antibody, cetuximab, for gastrointestinal cancer.

Parikh et al. have answered a clinically meaningful question.^1^ Even though, the biweekly (every 2 weeks) dosing of anti-epidermal growth factor receptor (anti-EGFR) monoclonal antibody cetuximab was being offered by providers and institutions alike to synchronize with most of our gastrointestinal (GI) oncology regimens, it was still not widely adopted (Fig. 1). Weekly administration of the 250 mg/m^2^ (following an initial loading dose of 400 mg/m^2^) is still the frequent dosing seen in practice and clinical trials. Some of the initial reports and suggestions to consider a biweekly regimen especially for patients with colorectal cancer date back to more than a decade ago.^2,3^ However, when this study was conducted, this was still an unmet need; a question that had not been formally answered. It is surprising to note that official United States Food and Drug Administration (US FDA) approval for the biweekly dosing of cetuximab was not till April 6, 2021.^4^

**Figure 1.**
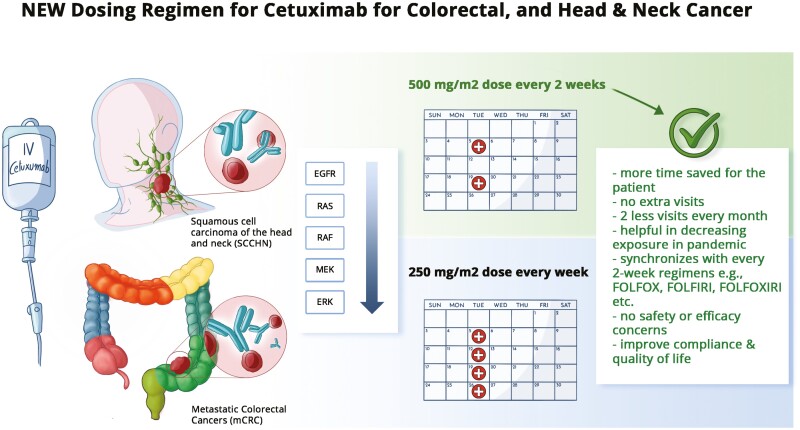
Advantages of biweekly versus weekly administration of cetuximab for patients with metastatic colorectal cancer (mCRC), and squamous cell carcinoma of the head and neck (SCCHN).

This study alongside recent pharmacokinetics data shows that the 500 mg/m^2^ dosing of intravenous (IV) cetuximab done every 2 weeks achieves similar drug exposures and clinical outcomes as compared to the 250 mg/m^2^ weekly dosing of the anti-EGFR agent. The pharmacokinetic modeling experiments and the efficacy that is seen is not just limited to patients with colorectal cancer but also patients with squamous cell carcinoma of the head and neck (SCCHN), where this agent is often used as a radiation sensitizer.^5,6^ Moreover, there is no added toxicity of giving the higher, less frequent dosing of cetuximab when compared with using the weekly regimen.

This meta-analysis is timely and backs up the US FDA approval of the biweekly dosing. It offers multiple advantages (Figure 1). It is a practical issue for patients with metastatic colorectal cancer receiving biweekly regimens like FOLFOXIRI, FOLFOX, or FOLFIRI with which this agent is usually paired with. Additionally, for patients switching to more so a maintenance regimen of anti-EGFR alone or with 5-fluourouracil (5-FU) chemotherapy, that would be 2 extra visits every month. In the middle of a pandemic as we limit exposures of patients who are already immunocompromised to begin with and at higher risk of morbidity and mortality from SARS-CoV-2 infection, minimizing an additional two visits every month is of great value.^7^ It also takes a huge burden off the system and oncology clinics who are already struggling to meet the needs of the current volume of patients. The biweekly dosing would also be more cost effective by limiting additional visits and infusion chair times.

In summary, while this study is not about a novel drug or regimen, it needs to be highlighted since this important work helps answer a clinically meaningful and practically relevant question of using cetuximab with an approved every other week dosing without compromising the efficacy and/or causing safety issues. This would also have bearing for other combination regimens, eg the BEACON regimen of using the anti-EGFR agent with a *BRAFV600E*-inhibitor for patients with colorectal cancer.^8^ Broad knowledge and adoption of less frequent dosing would be something that would be well received by oncologists, as well as patients and caregivers alike.
